# A rare long-term undetected pheochromocytoma leading to Takotsubo syndrome in an older male patient: a case report

**DOI:** 10.1186/s12902-020-00578-5

**Published:** 2020-06-23

**Authors:** Min Chen, Tong Zhao, Guoping Chen, Shenjiang Hu

**Affiliations:** 1 Department of Endocrinology, the First Affiliated Hospital, College of Medicine, Zhejiang University, #79, Qingchun Road, Zhejiang, 310003 Hangzhou China; 2 Department of Cardiology, the First Affiliated Hospital, College of Medicine, Zhejiang University, #79, Qingchun Road, Zhejiang, 310003 Hangzhou China

**Keywords:** Takotsubo syndrome, Pheochromocytoma, Cerebral hemorrhage, Differential diagnosis, Case report

## Abstract

**Background:**

Takotsubo syndrome is an uncommon, acute, and reversible cardiomyopathy that occurs primarily in postmenopausal females. The clinical presentation of the syndrome resembles acute coronary syndrome, but coronary angiography reveals no obstructive coronary artery disease. Rarely, a catecholamine surge due to pheochromocytoma may induce Takotsubo syndrome. The clinical features of pheochromocytoma include paroxysmal hypertension, headache, palpitations, and profuse sweating. However, owing to the episodic, rather than continued, symptoms and signs of pheochromocytoma, its timely diagnosis poses a challenge for clinicians. Here, we report a rare case of long-term undetected pheochromocytoma leading to Takotsubo syndrome in an older male patient.

**Case presentation:**

A 70-year-old man presented with paroxysmal chest distress and chest pain. Examinations revealed acute coronary syndrome with normal coronary arteries, heart failure, reversible left ventricular regional wall motion abnormalities, labile blood pressure, a giant left adrenal mass, and extremely high levels of metanephrine and normetanephrine. Clinical manifestations, laboratory reports, and imaging findings suggested a diagnosis of Takotsubo syndrome caused by pheochromocytoma. Supportive therapy, administration of alpha- adrenergic receptor blockers, and left adrenal mass resection resolved the patient’s symptoms. A histological examination confirmed the presence of pheochromocytoma. We reviewed his history of midbrain hemorrhage 6 years prior and found a mass in the left adrenal region by reviewing the computed tomography images of the lung that were also taken 6 years prior, on which the pheochromocytoma was evident.

**Conclusions:**

Our case illustrates the importance of understanding the link between pheochromocytoma and Takotsubo syndrome. A diagnosis of pheochromocytoma-induced Takotsubo syndrome should be considered during the differential diagnosis of acute coronary syndrome, especially in patients with labile blood pressure and normal coronary angiography findings; meanwhile, assessments of catecholamines and its metabolites and abdominal computed tomography scan should be performed at the right time. Clinicians should also be alert to potential pheochromocytoma in patients with unexplained cerebral hemorrhage, even in the absence of symptoms of catecholamine excess.

## Background

Takotsubo syndrome, also known as “broken heart syndrome”, is an increasingly recognized acute and reversible cardiomyopathy that usually follows intense physical or emotional stress and is associated with an elevated catecholamine level [1]. The clinical presentation of the syndrome resembles acute coronary syndrome (ACS), but coronary angiography reveals no obstructive coronary artery disease [1, 2]. Takotsubo syndrome is rarely induced by a catecholamine surge due to pheochromocytoma [3]. Typical clinical features of pheochromocytoma include paroxysmal hypertension, headache, palpitations, and profuse sweating. However, pheochromocytoma usually results in a paroxysmal increase in catecholamine levels, which poses a challenge for clinicians to diagnose in time because of the episodic, rather than continued, symptoms and signs. We report a rare long-term undetected pheochromocytoma leading to Takotsubo syndrome in an elderly male patient.

## Case presentation

A 70-year-old Chinese man was admitted to our hospital complaining of a 12-h history of paroxysmal chest distress and chest pain. He had a past medical history of midbrain hemorrhage 6 years ago. He denied a history of hypertension, diabetes, paroxysmal headache, palpitations, or profuse sweating. He denied a history of drug abuse or recent intense emotional stress. His family history was unremarkable. The patient, without obvious inducement, suddenly experienced chest tightness and pain while in bed at 1 a.m. The paroxysmal chest pain lasted about 1 h each time and was accompanied by a feeling of crushing in the precordial region, sweating, shortness of breath, orthopnea, and nausea. Upon admission, a physical examination revealed a blood pressure of 185/122 mmHg, a heart rate of 100 beats/min, and a respiratory rate of 32 breaths/min. A pulmonary examination showed diffuse moist rales in the lungs. The initial results of laboratory findings (Table 1) showed elevated white blood cell count, myocardial enzyme, and troponin I levels. The extremely high levels of brain natriuretic peptide and arterial blood gases suggested that the patient might have heart and respiratory failure. Electrocardiography findings suggested sinus tachycardia and mild ST-segment depression in leads V4-V6. A pulmonary computed tomography (CT) scan showed bilateral pulmonary edema and a massive lesion in the left adrenal area. Transthoracic echocardiography showed left ventricular regional wall (apical wall and mid septal wall) motion abnormalities with an ejection fraction of 34%. The clinical diagnosis was ACS, heart failure, and respiratory failure. The patient was given a venturi mask for oxygen inhalation, morphine for pain relief and vasodilation, furosemide for diuresis, isosorbide nitrate for coronary artery dilation, aspirin combined with clopidogrel for antiplatelets, low molecular weight heparin for anticoagulation, and atorvastatin for lipid- lowering.

**Table 1:** Initial laboratory test results

Parameter (unit)	Result	Reference value
White blood cell count (× 10^9^/L)	26.1	4–10
Troponin I concentration (ng/mL)	10.85	< 0.060
CK (U/L)	1186	38–174
CK − MB (U/L)	77	2–25
Brain natriuretic peptide (pg/mL)	> 9000	< 376
Arterial blood pH	7.29	7.35–7.45
PaO_2_ (mmHg)	57.3	80.0–110.0
PaCO_2_ (mmHg)	38.6	35.0–45.0
Arterial lactate (mmol/L)	4.16	0.5–2.2
serum potassium (mmol/L)	4.39	3.5–5.2
Serum calcium (mmol/L)	2.24	2.03–2.54
Serum creatinine (μmol/L)	113.9	59–104

*CK* Creatine kinase, *PaO*_*2*_ Arterial partial pressure of oxygen, *PaCO*_*2*_ Arterial partial pressure of carbon dioxide.

The next morning, the patient experienced severe chest distress and shortness of breath. His blood pressure fluctuated from 200/100 mmHg to 55/30 mmHg. An electrocardiogram showed ST- segment elevation in leads II, III, aVF, and V4–V6 and paroxysmal ventricular tachycardia. The levels of myocardial enzyme and troponin I increased progressively. Coronary angiography was performed to investigate possible acute myocardial infarction; however, the coronary arteries appeared normal. Based on the coronary angiography results, we stopped the antiplatelet and anticoagulation treatments.

**Fig. 1:**
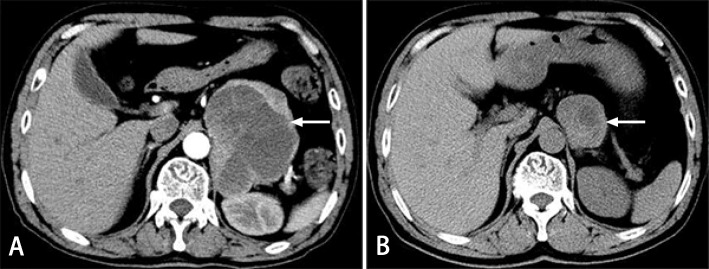
Abdominal computed tomography (CT) scan (**a**) shows a 9.6 × 8.3 cm left adrenal mass with an inhomogeneous density; lung CT scan (**b**) shows a 4.4 × 4.0 cm mass in the left adrenal area

At this time, we noticed that the patient had a left adrenal area mass that was accidentally discovered on a CT scan of the lung. An abdominal and pelvic CT scan revealed a left adrenal mass with an inhomogeneous density measuring 9.6 × 8.3 cm, and a contrast-enhanced CT scan demonstrated heterogeneous enhancement and multiple internal septum changes (Fig. 1(a)). The apparent ACS and labile blood pressure associated with the enormous left adrenal mass raised the suspicion of pheochromocytoma, which was further supported by markedly elevated levels of catecholamine metabolites, plasma normetanephrine> 10,000 pg/mL (reference value, 19–121 pg/mL), and plasma metanephrine levels of 6356.17 pg/mL (reference value, 14–90 pg/mL). Subsequent laboratory tests were performed, and the following results were obtained: 24-h urinary epinephrine, 139 μg (reference value, 0–20 μg); 24-h urinary norepinephrine, 377 μg (reference value, 15–80 μg); serum calcitonin< 2.0 pg/mL (reference value, 0–8.4 pg/mL); and parathyroid hormone 27.8 pg/mL (reference value, 12–65 pg/mL). Intravenous urapidil hydrochloride was administered for hypertension, while endotracheal intubation and mechanical ventilation were initiated for respiratory support. His blood pressure and levels of myocardial enzyme and troponin I gradually recovered to normal. On day 5 of admission, a repeat echocardiogram showed no obvious segmental abnormalities in the left and right ventricular wall, with recovery of the ejection fraction to 63% and normal systolic function.

**Fig. 2:**
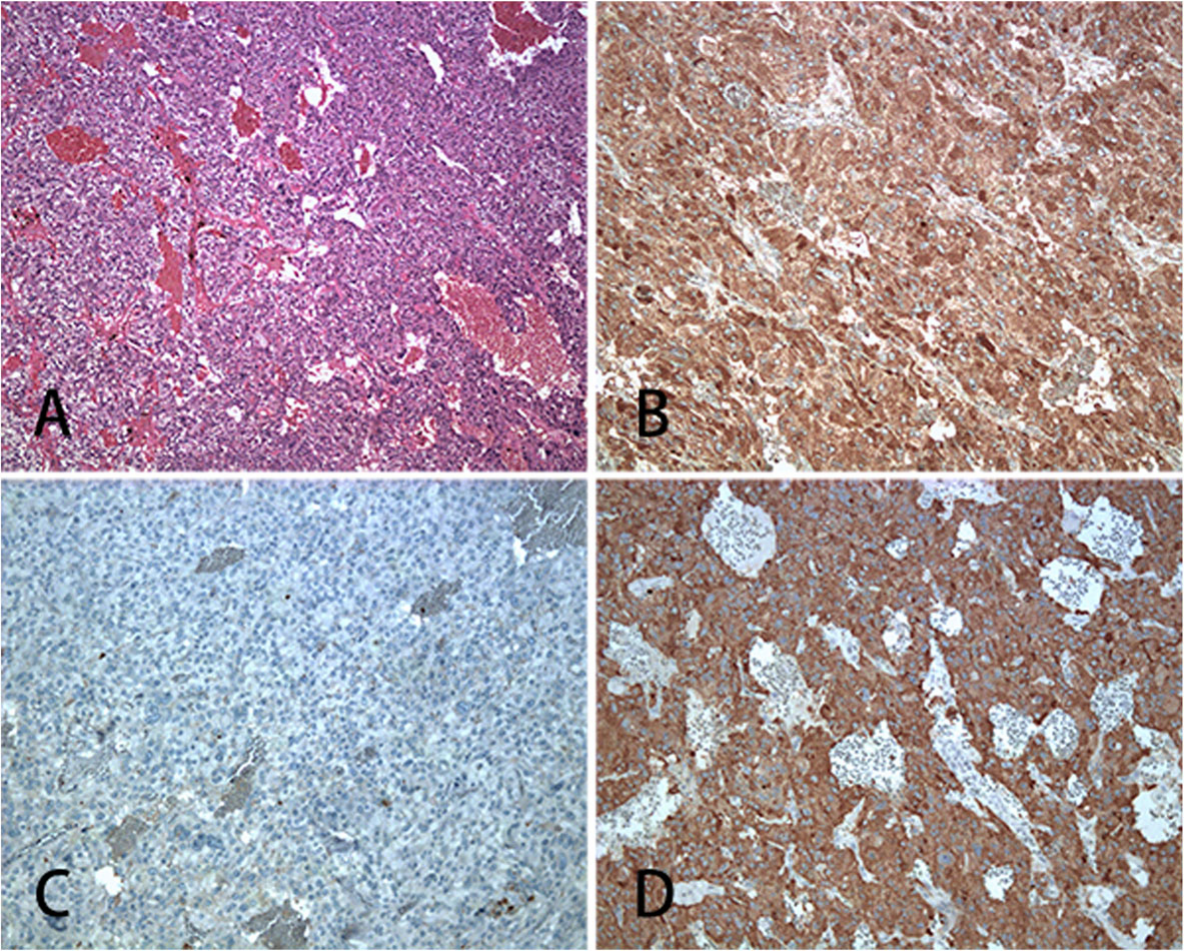
Hematoxylin and eosin staining of the adrenal tumor, Magnification × 100 (**a**); immunohistochemistry positive for chromogranin (**b**), synaptophysin (**d**), and negative for cytokeratin (**c**)

After the patient’s condition improved, some examinations were carried out. Plasma aldosterone/renin activity ratio, sex hormones, and 17-hydroxyprogesterone tests were in the normal range; No abnormalities were found in 24-h urinary free cortisol excretion and 1 mg overnight dexamethasone suppression test. Thyroid ultrasound and cranial magnetic resonance imaging (MRI) results were unremarkable. Considering that contrast-enhanced CT of the chest and abdomen and cranial MRI scans did not indicate suspicious metastases or extra-adrenal lesions, we decided to perform a left adrenalectomy. Terazosin was administered and titrated over approximately 3 weeks to provide an adequate alpha blockade before the surgical procedure. A histological examination of the adrenal gland mass confirmed that it was a pheochromocytoma (Fig. 2). Postoperatively, the patient’s condition drastically improved. His blood pressure normalized, and he experienced no chest distress or pain. During the 9 months of regular follow-up, he had no symptoms and was in good condition. His levels of metanephrine, normetanephrine, parathyroid hormone, serum calcium, and calcitonin were in the normal range. His electrocardiogram and echocardiogram findings were normal. No suspicious lesions were found on the abdominal and pelvic CT enhanced scans.

**Fig. 3:**
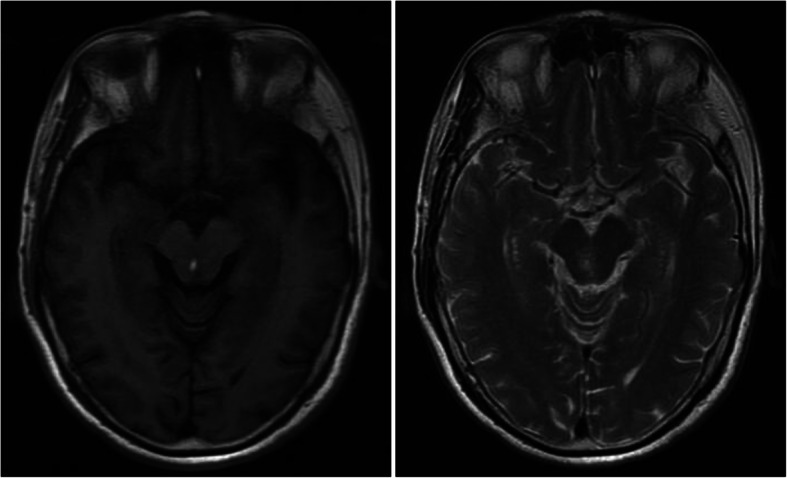
An abnormal patch signal is shown in the midbrain on cranial magnetic resonance imaging, high signal intensity on the T2-weighted imaging, and higher signal on the T1-weighted imaging

At this point, we reviewed his past medical history of a midbrain hemorrhage 6 years prior. The patient was referred to the hospital with a 7-day history of suddenonset diplopia and dizziness. A cranial CT revealed a high-density lesion in the midbrain. An abnormal patch signal was found in the midbrain on cranial MRI, high signal intensity on T2-weighted imaging, a higher signal intensity on T1-weighted imaging, a slightly higher signal intensity on a diffuse weighted imaging sequence, and a heterogeneous intensity on a fluid-attenuated inversion recovery sequence. The results of the cranial MRI suggested a small midbrain hemorrhage (Fig. 3). No abnormalities were found on CT angiography (CTA) of the vertebrobasilar artery. After medical treatment, the symptoms of diplopia and dizziness gradually improved. At that time, a lung CT revealed a mass (4.4 × 4.0 cm) in the left adrenal area [Fig. 1(b)]. We believed that the left adrenal mass that existed six years prior was the pheochromocytoma currently confirmed by histological examination.

## Discussion and conclusions

Pheochromocytoma is a catecholamine-secreting tumor arising from chromaffin cells in the adrenal medulla. Its classic symptoms include episodic headache, palpitations, and profuse sweating accompanied by paroxysmal or sustained hypertension. Pheochromocytoma usually results in a paroxysmal increase in catecholamine levels— sometimes massively elevated— which may trigger serious cardiocerebrovascular complications such as Takotsubo syndrome [4], heart failure [5], cerebral hemorrhage [6], and sudden death [7].

Takotsubo syndrome is an uncommon and acute transient reversible cardiac disease entity that usually follows intense physical or emotional stress and is more likely to occur in postmenopausal females [4]. The word *takotsubo* is used to represent the left ventricular profile during systole in patients with clinical characteristics of myocardial infarction, meanwhile, coronary angiography reveals normal coronary arteries or coronary arteries with minimal atherosclerotic changes. The clinical manifestations, electrocardiographic changes, and myocardial infarction biomarkers of this syndrome often resemble those of ACS [8]. The main characteristic of Takotsubo syndrome includes a regional left ventricular wall motion abnormality (LVWMA) with a specific round pattern leading to significant ballooning of the left ventricle during systole. In patients with Takotsubo syndrome, left ventricular dysfunction is generally reversible and almost completely resolves within hours to weeks [8].

Our patient with pheochromocytoma had an unusual presentation (apparent ACS, reversible LVWMA, no coronary artery obstruction), for which Takotsubo syndrome was diagnosed. The diagnosis of pheochromocytoma-induced Takotsubo syndrome was supported by the pathological examination of an adrenal mass, massively increased metanephrine and normetanephrine levels, and rapid LVWMA recovery. Review of the patient’s history of midbrain hemorrhage— based on the sudden onset of symptoms of oculomotor nerve damage, the curative effect of medical treatment, and the results of intracranial imaging— led to the diagnosis of a small midbrain hemorrhage. It is worth noting that we found a mass in the left adrenal region by review of the patient’s lung CT images taken 6 years prior. Unfortunately, the physicians did not notice the adrenal mass at that time. We believe that the left adrenal mass that existed 6 years prior was the pheochromocytoma noted in this case. However, due to the absence of typical symptoms of catecholamine excess 6 years prior, no adrenal imaging or test of catecholamines and its metabolites was performed. This resulted in a 6-year delay in the diagnosis. The adrenal mass further increased during the 6 years and was later confirmed as pheochromocytoma by postoperative pathological analysis.

The pathophysiological mechanism of Takotsubo syndrome resulting from pheochromocytoma is not well understood. Pheochromocytoma with extremely elevated catecholamine levels may be a strong physical trigger factor for Takotsubo syndrome induction, most likely through hyperactivation of the sympathetic nervous system, including cardiac sympathetic nerve terminal disruption with norepinephrine seethe and spillover [8]. Catecholamines play a vital role in triggering Takotsubo syndrome. Clinical researches have reported that mental stress can decrease ejection fraction, rarely causing regional wall motion abnormalities with increased catecholamine levels [9]. Furthermore, several diseases such as subarachnoid hemorrhage [10] and pheochromocytoma [11] presenting with wall motion abnormalities and reduced ejection fraction are associated with high concentrations of catecholamines. In the presence of Takatsubo syndrome, catecholamine concentrations are extremely high and remain elevated for 7–9 days [12]. The extremely high levels of metanephrine and normetanephrine in this patient also suggested that catecholamine may be a trigger factor for pheochromocytoma-induced Takotsubo syndrome.

Intracerebral hemorrhages can be caused by a cerebral aneurysm, arteriovenous malformations, or hypertension. Some less-common causes, such as eclampsia, cerebral venous thrombosis, drug abuse, and pheochromocytoma, can also result in intracerebral hemorrhage [6]. Neither MRI nor CTA revealed an obvious underlying cause of the hemorrhage; we speculated that the midbrain hemorrhage that occurred 6 years prior might be related to the pheochromocytoma in our patient.

As pheochromocytoma can be fatal, it is challenging for clinicians to diagnose it early. Timely diagnosis may lead to targeted drug therapy and tumor removal, and may substantially improve prognosis. Otherwise, an undetected pheochromocytoma may suddenly secrete a large amount of catecholamine, which may cause severe cardiocerebrovascular complications. There is little evidence of a link between long-term undetected pheochromocytoma and Takotsubo syndrome. We lack direct evidence to conclude that a person without Takotsubo syndrome susceptibility factors (emotional stress, postmenopausal status, etc.) could develop Takotsubo syndrome owing to a long-term undetected pheochromocytoma. Six years prior, if the adrenal mass during hematencephalon had been found, Takotsubo syndrome may have been avoidable.

The treatment of pheochromocytoma-induced Takotsubo syndrome mainly consists of supportive therapy, preoperative medical management, and tumor resection. Recently, a retrospective study of case reports revealed that complications rates (such as cardiogenic shock and heart failure) were higher in patients with pheochromocytoma-induced Takotsubo syndrome than in those with primary Takotsubo syndrome [3]. Sometimes, extracorporeal life support, including extracorporeal membrane oxygen or left ventricular assist treatment, is required in supportive therapy [13, 14]. Alpha- adrenergic receptor blockers are the first choice in the preoperative management to prevent perioperative cardiocerebrovascular complications. It should be noted that most patients with primary Takotsubao syndrome are usually treated with beta- adrenergic receptor blockers; however, selective blockade of beta- adrenergic receptors may cause hyperactivation of alpha- adrenergic receptors in patients with pheochromocytoma and further aggravate the illness. Therefore, patients with suspected pheochromocytoma-induced Takotsubo syndrome should be administered simultaneous alpha- and beta- adrenergic receptor blockers [15].

Notably, Takotsubo syndrome usually occurs in postmenopausal females. A recent Takotsubo syndrome case series analysis uncovered that almost 90% of all patients were females, aged 58–75 years [16]. Little is known about the reason for the predominance of older females, but it makes us consider whether withdrawal from estrogens is a risk factor of its pathogenesis. Clinical researches have revealed that chronic estrogen supplementation attenuated blood pressure, glucocorticoid, and catecholamine responses to mental stress [17], as well as catecholamine-mediated vasoconstriction [18]. However, our patient was male and did not meet the profile of a typically susceptible individual. There is no direct evidence that the huge pheochromocytoma increased our patient’s susceptibility.

Our findings suggest that the link between pheochromocytoma and Takotsubo syndrome is importance. A diagnosis of pheochromocytoma-induced Takotsubo syndrome should be considered in the differential diagnosis of ACS, especially in patients with labile blood pressure and normal coronary angiography findings; meanwhile, catecholamines test and abdominal CT scans should be performed promptly. Clinicians should also be alert to the possibility of pheochromocytomas in the evaluation of unexplained cerebral hemorrhage, even in the absence of symptoms of catecholamine excess.

## Data Availability

The datasets used and analysed during the current study are available from the corresponding author on reasonable request.

## Abbreviations

ACS: Acute coronary syndrome
CK: Creatine kinase.
CT: Computed tomography.
MRI: Magnetic resonance imaging.
LVWMA: Left ventricular wall motion abnormality.
